# Prioritization of intervention domains to prevent cardiovascular disease: a country-level case study using global burden of disease and local data

**DOI:** 10.1186/s12963-023-00301-1

**Published:** 2023-01-26

**Authors:** Nick Wilson, Christine Cleghorn, Nhung Nghiem, Tony Blakely

**Affiliations:** 1 Department of Public Health, University of Otago, Wellington, New Zealand; 2 School of Population and Global Health, The University of Melbourne, Parkville, VIC Australia

**Keywords:** Cardiovascular disease, Risk factors, Burden of disease, Prioritization, Health economic evaluation, DALYs, Preventive interventions

## Abstract

**Aim:**

We aimed to combine Global Burden of Disease (GBD) Study data and local data to identify the highest priority intervention domains for preventing cardiovascular disease (CVD) in the case study country of Aotearoa New Zealand (NZ).

**Methods:**

Risk factor data for CVD in NZ were extracted from the GBD using the “GBD Results Tool.” We prioritized risk factor domains based on consideration of the size of the health burden (disability-adjusted life years [DALYs]) and then by the domain-specific interventions that delivered the highest health gains and cost-savings.

**Results:**

Based on the size of the CVD health burden in DALYs, the five top prioritized risk factor domains were: high systolic blood pressure (84,800 DALYs; 5400 deaths in 2019), then dietary risk factors, then high LDL cholesterol, then high BMI and then tobacco (30,400 DALYs; 1400 deaths). But if policy-makers aimed to maximize health gain and cost-savings from specific interventions that have been studied, then they would favor the dietary risk domain (e.g., a combined fruit and vegetable subsidy plus a sugar tax produced estimated lifetime savings of 894,000 health-adjusted life years and health system cost-savings of US$11.0 billion; both 3% discount rate). Other potential considerations for prioritization included the potential for total health gain that includes non-CVD health loss and potential for achieving relatively greater per capita health gain for Māori (Indigenous) to reduce health inequities.

**Conclusions:**

We were able to show how CVD risk factor domains could be systematically prioritized using a mix of GBD and country-level data. Addressing high systolic blood pressure would be the top ranked domain if policy-makers focused just on the size of the health loss. But if policy-makers wished to maximize health gain and cost-savings using evaluated interventions, dietary interventions would be prioritized, e.g., food taxes and subsidies.

## Introduction

Ischemic heart disease (IHD) is the highest ranked cause of health loss in New Zealand (NZ), when considering death and disability combined [1]. The other key component of cardiovascular disease (CVD) is stroke, which is ranked fifth in importance for health loss (i.e., albeit behind low back pain, chronic obstructive pulmonary disease and falls). IHD is the leading cause of death in the country, followed by stroke [1]. The total annual CVD burden for NZ is estimated at 11,900 deaths and 183,000 disability-adjusted life years (DALYs), or 15.1% of all DALYs [2]. In addition, CVD is an important contributor to health loss for Māori (Indigenous population) and it contributes to health inequities in NZ in terms of both ethnicity [3–5] and socioeconomic position [6].

CVD is also expensive with an estimated annual cost to the health system of US$2.3 billion [7] (~ NZ$3.3 billion). In addition, there is the annual loss of income to NZ citizens from CVD, estimated at US$427 million (15.6% of all disease-related income loss; and far ahead of cancer-related income loss at US$122 million) [8]. The high costs of CVD to the NZ Government are a particularly important consideration when the country’s health system is chronically fiscally constrained and has the recently added stressors associated with the Covid-19 pandemic.

CVD has also been given high priority by NZ stakeholders in a multi-criteria decision analysis to prioritize non-communicable diseases for research funding decision-making [9]. That is, coronary heart disease was in the top priority group, along with back and neck pain, and diabetes mellitus. Stroke was in the next highest priority group, along with “dementia and Alzheimer’s disease.” Furthermore, there is substantial scope for CVD prevention given that there are so many CVD prevention interventions available and which can be intensified [10, 11]. Many of these CVD preventive interventions can also contribute to health gain in other domains, e.g., reducing tobacco use can reduce both CVD and a wide range of cancers. While it may be more rational for policy-makers to focus on major risk factors for health loss (such as diet and smoking) as opposed to disease domains (such as CVD), we suspect that the disease domain focus is useful for policy-makers to explain to the public. For example, stating that “we plan to prevent heart disease” may be more understandable than (or at least a useful adjunct to) “we plan to control risk factors that cause the most health loss.”

The NZ health system (like many countries) has made substantial progress in preventing CVD with such measures as ongoing enhancements to tobacco control (with even more substantive declines in smoking recently [12]). There have also been ongoing improvements in the provision of preventive pharmacotherapy. The assessing of absolute CVD risk for CVD risk management (i.e., prioritizing preventive pharmacotherapy and counseling by overall 5-year risk of a CVD event), has been promoted to clinicians for a long time [13], albeit with this approach still not always dominating in practice [14]. There is also evidence for successful campaigns to increase the use of preventive pharmacotherapy, e.g., use of lipid-lowering statins in Māori [15].

Given this background, we aimed in this study to prioritize CVD risk factor domains for NZ when considering the size of the health loss and also the potential health gains and health economic benefits of preventive interventions.

## Methods

We extracted risk factor data for CVD in NZ from the Global Burden of Disease (GBD) Study using the “GBD Results Tool” and using the disease category of “B.2 Cardiovascular diseases” [2]. This data source was selected on the basis of the high quality of the risk factor analysis as detailed in these recent publications [10, 11, 16].

These risk factor domains were then ranked based on consideration of the highest CVD-related health burden as measured by disability-adjusted life years (DALYs), i.e., a composite of health loss from premature death and disability. We then took the top five risk factor domains for CVD for further consideration, with just five being selected on the basis of encouraging a more strategic focus by policy-makers. Such a focus seems needed given that the NZ Government does not currently have any systematic strategic plan for non-communicable disease prevention or for CVD prevention, and does not routinely use health economic evidence for prioritizing public health interventions (with prioritizing by the agency PHARMAC for pharmaceuticals being an exception [17]).

We then further identified the top priority risk factor domains by additionally considering published evaluations of domain-specific interventions that delivered the highest health gains and cost-savings. To inform such prioritization, we conducted literature searches to identify relevant health economic evaluation studies. The search method used was identical to a previous search used to identify NZ-relevant studies published in the peer-reviewed journal literature between January 1, 2010, and October 8, 2017 (search details described elsewhere: [18]). In summary, we searched for NZ-related studies with the following metrics: cost per quality-adjusted life-year or disability-adjusted life-year or health-adjusted life-year or life-year (QALY/DALY/HALY/LY), to cover October 1, 2017, to December 31, 2021.

## Results

Based on the size of the CVD health burden in DALYs, the five top prioritized risk factor domains out of all those detailed in the GBD Study were: high systolic blood pressure (84,800 DALYs; 5400 deaths in 2019), then dietary risk factors, then high LDL cholesterol, then high BMI and then tobacco (30,400 DALYs; 1400 deaths in 2019) (Table 1). For these five risk factors, the same ranking order was apparent in terms of number of deaths. Nevertheless, given the overlapping 95% uncertainty intervals in both DALYs and deaths, this ranking can only be considered approximate.

**Table 1:** CVD burden in 2019 for NZ attributable to specific risk factors and ranked by the number of disability-adjusted life years (DALYs; for all ages, both sexes (95% uncertainty intervals), GBD data extracted using the “GBD Results Tool”)

Risk factor*	CVD deaths		DALYs (ranked)	
	Count	Proportion (%**)	Count	Proportion (%**)
High systolic blood pressure	5400 (4210 to 6470)	45.2	84,800 (71,400 to 97,700)	46.3
Dietary risk factors—all (see also below for specific components)	3970 (3180 to 4810)	33.3	62,400 (51,000 to 74,900)	34.1
High LDL cholesterol	3330 (2300 to 4530)	27.9	51,200 (40,000 to 64,600)	28.0
High body-mass index (BMI)	1940 (1130 to 2850)	16.3	40,100 (25,500 to 56,300)	21.9
Tobacco (including secondhand smoke***)	1400 (1270 to 1520)	11.7	30,400 (28,000 to 32,900)	16.6
High fasting plasma glucose	2000	17.0	27,000	14.7
Kidney dysfunction	1200	10.1	15,400	8.38
Non-optimal temperature (just too low for NZ data and not including excessive temperature as in heat waves)	862	7.21	11,400	6.22
Low physical activity	673	5.63	7950	4.34
Alcohol use	103	0.87	4760	2.60
Lead exposure	258	2.16	3960	2.16
Air pollution in the form of ambient particulate matter pollution	172	1.44	3190	1.74
**More specific risk factors—diet**				
Diet low in whole grains	1010	8.48	16,000	8.73
Diet high in red meat	814	6.82	14,400	7.84
Diet low in legumes	878	7.35	13,600	7.44
Diet high in trans fatty acids	502	4.21	7710	4.21
Diet high in sodium	377	3.16	6920	3.78
Diet low in dietary fiber	377	3.15	5720	3.12
Diet low in fruits	296	2.48	5050	2.76
Diet low in vegetables	323	2.70	4880	2.66
Diet high in processed meat	247	2.07	4370	2.39
Diet low in seafood omega-3 fatty acids	201	1.69	3030	1.66
Diet low in polyunsaturated fatty acids	192	1.61	2990	1.63
Diet high in sugar-sweetened beverages	104	0.87	1650	0.90
Diet low in nuts and seeds	137	1.15	1620	0.88

*Most of these risk factors are not independent of one another. For example, the blood pressure risk factor, the high LDL cholesterol risk factor and high BMI risk factor will be partly mediated via dietary risk factors. Nevertheless, a few of the risk factors (e.g., air pollution) may be largely independent of the other listed risk factors.

**This is the proportion out of the total of 11,900 deaths and 183,000 DALYs attributed to CVD in NZ in 2019 (with 79.6% of the total DALYs being attributed to named risk factors in the GBD).

***Of the tobacco group, 11% of the deaths and DALYs were attributed to secondhand smoke exposure.

Values rounded to three meaningful digits.

Within the dietary risk grouping, the three CVD risk factors associated with the highest DALYs were: diet low in whole grains, then diet high in red meat, and then diet low in legumes (Table 1). All of these CVD risk factors had higher rankings than the ones of low physical activity and alcohol use.

When considering specific interventions generating health gain and being cost-saving or cost-effective (or not) in the NZ setting for these five priority domains, a total of 22 relevant peer-reviewed publications were identified (published since January 1, 2010; Table 2). In terms of the size of health gain and cost-savings from these, the highest impact intervention was a dietary one, i.e., a combined fruit and vegetable (F&V) subsidy plus a sugar tax (Table 2). (While a “radical” intervention, it is important to note that the interventions were designed to be cost-neutral to the consumer, with the net price of a standard basket of groceries unchanged by the tax-increases in price offset by subsidy-decreases.) This produced estimated lifetime savings of 894,000 health-adjusted life years and health system cost-savings of US$11.0 billion (~ NZ$16.4 billion; 3% annual discount rate). Behind this in impact were a sugar tax, then a salt tax with F&V subsidy, then a saturated fat tax with F&V subsidy, then a salt tax, and then a saturated fat tax. All these six dietary interventions were more impactful (greater health gain and cost-savings) than the highest impact tobacco control intervention: a sinking lid on tobacco sales.

**Table 2:** Top five risk factors for CVD health loss ordered by decreasing size of health gain (through all diseases) and cost-savings from studied interventions in the NZ context

Risk factor (top 5 from Table 1)	Highest impact health gain from an intervention	Highest impact cost-saving* from an intervention	Details of the relevant NZ health economic publications (with all specific health gain and cost values given being for lifetime impacts at an annual discount rate of 3% and a threshold for defining "cost-effective" used**)
Dietary risk factors	894,000 HALYs gained (a combined fruit and vegetable (F&V) subsidy plus a sugar tax) [19]	US$ 11.0 billion saved (US$ 2018) (as per the intervention in the column to the left)	*Cost-saving:* (i) Various combinations of food taxes and subsidies [19]. The highest impact intervention was a combined fruit and vegetable (F&V) subsidy plus a sugar tax. Behind this in impact were a sugar tax, then a salt tax with F&V subsidy, then a saturated fat tax with F&V subsidy, then a salt tax, and then a saturated fat tax. Higher per capita health gains for Māori vs non-Māori were identified
			(ii) Adoption of climate-friendly eating patterns [20] (the model included multiple CVD risk factors list in Table 1 including: red meat, sugar-sweetened beverages (SSBs), and sodium as well as low intake of fruit, vegetables, and polyunsaturated fat). But the economic analysis did not include the intervention costs associated with achieving these dietary pattern changes and it was assumed that the whole population shifted eating patterns
			(iii) A cap on the size of single servings for SSBs [21]
			*Cost-effective:* A multicomponent through-school physical activity and nutrition program (“Project Energize”) [22]
			*Comment:* There is some overlap with this dietary risk factor grouping in this table and interventions to reduce BP, lower LDL cholesterol and to lower BMI (as detailed elsewhere in this table). Some of the cost-saving interventions capture non-CVD health benefits (e.g., preventing diet-related cancers, diabetes etc.)
High systolic blood pressure (BP)	453,000 HALYs gained (salt tax) [19]	US$ 5.90 billion saved (US$ 2018) (salt tax) [19]	*Cost-saving:* (i) Three publications each involving multiple different dietary salt reduction interventions (including salt substitution, salt tax, UK style interventions etc.) [23–25]; (ii) salt tax [19]
			*Cost-effective:* (i) A “soft regulation” national policy for dietary sodium reduction that combines targeted industry agreements, government monitoring, and public education (international study with NZ data) [26]
			(ii) Double therapy (an antihypertensive and a statin) and antihypertensive alone by clinician-assessed absolute risk level (cost-effective for nearly all risk levels in the middle-aged male age-group studied) [27]
			*Comment:* There is some overlap with these BP interventions with those in the dietary risk factor grouping (elsewhere in this table) given that some of the latter will lower sodium intake and increase potassium intake
High LDL cholesterol	436,000 HALYs gained (saturated fat tax) [19]	US$ 5.87 billion saved (US$ 2018) (saturated fat tax) [19]	*Cost-saving:* Saturated fat tax [19]
			*Cost-effective:* Double therapy (a statin and antihypertensive) and statin alone by clinician-assessed absolute risk level (at least in middle-aged males) [27]
			*Comment:* There is some overlap with this risk factor grouping and the dietary interventions detailed elsewhere in this table (many of which would reduce dietary intakes of saturated fat and increase intakes of polyunsaturated fat)
Tobacco use	282,000 QALYs gained (from a sinking lid on supply) [28]	NZ$ 5.43 billion saved (NZ$ 2011) (~ US$ 4.07 in US$ 2018) (from a sinking lid on supply) [28]	*Cost-saving:* (i) Two tobacco tax increase studies [29, 30]
			(ii) Reduced retail access [31]
			(iii) Five endgame interventions (including impact on access and supply etc.) [28]. Higher per capita health gains for Māori vs non-Māori were identified
			(iv) Pharmacy-only sales and pharmacist counseling [32]
			(v) Promotion of the Quitline for smoking cessation [33]
			(vi) Promoting smartphone apps for smoking cessation [34]
			(vii) Two studies [35, 36] on permitting ready access to e-cigarettes (albeit this has now largely occurred in NZ)
			*Not cost-effective:* Exercise counseling intervention to enhance smoking cessation [37]
			*Comment:* All the cost-saving studies detailed above capture CVD-related health benefits but also the benefits of preventing 14 other tobacco-related diseases. In one tobacco tax intervention study that identified how the QALYs gained were distributed, 16.6% were from CVD prevention and the majority were from chronic respiratory disease prevention (Table S6 in Blakely et al. [29])
High body-mass index (BMI)	250 QALYs gained (from the weight-loss counseling intervention applied to 21.6% of the eligible population [38]). A total was not calculated for any scaled up form of “Project Energize”	No cost-saving intervention identified	*Cost-saving:* Nil
			*Cost-effective:* A multicomponent through-school physical activity and nutrition program (“Project Energize”) [22]. But this was not cost-effective in some sensitivity analyses, e.g., 5% decay per annum in BMI change after the first 5 years
			*Not cost-effective:* (i) Weight-loss dietary counseling by nurses in primary care [38]; (ii) Mass media promotion of smartphone apps for weight loss [39]
			*Comment:* There is considerable overlap with this BMI risk factor grouping and the dietary intervention grouping (as detailed elsewhere in this table). We did not include four health economic studies of physical activity interventions [40–43], given that the evidence of the association between physical activity and BMI is not particularly strong

*That is, cost-saving from a NZ health system perspective at typically a 3% discount rate.

**With cost-effective being defined as up to the GDP per capita of NZ (NZ$45,000 in 2011 or ~ US$31,000) as per the standard BODE^3^ modeling approach for NZ analyses [44].

As some policy-makers may not consider population-level preventive interventions to be politically feasible, we also extracted individual-level interventions from the 22 peer-reviewed publications that were identified (Table 3). None of these were estimated to be cost-saving, in contrast to many of population-level interventions given in Table 2. Nevertheless, some were fairly cost-effective with the highest ranking one being in the joint risk factor domains of high blood pressure and high LDL cholesterol. That is, the use of double therapy (an antihypertensive and a statin) by clinician-assessed absolute risk level was estimated to gain a QALY for only NZ$ 1580 (i.e., in the highest risk stratum).

**Table 3:** Cost-effectiveness of individual-level interventions for preventing CVD from studied interventions in the NZ context (in descending order of cost-effectiveness)

Risk factor (top 5 from Table 1)	Intervention	Cost-effectiveness (incremental cost-effectiveness ratio [ICER])*	Further details and comments
High systolic blood pressure (BP) and high LDL cholesterol	Double therapy (an antihypertensive and a statin) by clinician-assessed absolute risk level	NZ$ 1580 per QALY gained in the > 20% in 5 years risk stratum (NZ$ 2011) (~ US$ 1160 in 2018)	Even in the lowest risk stratum (≤ 5% risk in 5 years), the cost per QALY was only NZ$ 25,500. These values were all just for middle-aged males [27]
High LDL cholesterol	Lipid-lowering therapy (informed by clinician-assessed absolute risk level)	NZ$ 3740 per QALY gained in the > 20% in 5 years risk stratum (NZ$ 2011) (~ US$ 2750 in 2018)	This compared with the ICER in the lowest risk stratum (≤ 5% risk in 5 years), of $43,500 (95% uncertainty interval [UI]: $22,400 to $73,700) [27]. These values were all just for middle-aged males. Of note was that the ICER was more favorable when double therapy was studied (see elsewhere in this table)
High systolic BP	Antihypertensive therapy (informed by clinician-assessed absolute risk level)	NZ$ 6470 per QALY gained in the > 20% in 5 years risk stratum (NZ$ 2011) (~ US$ 4760 in 2018)	This compared with the ICER in the lowest risk stratum (≤ 5% risk in 5 years), of NZ$ 62,400 (95%UI: 33,600 to 104,000) [27]. These values were all just for middle-aged males. Of note was that the ICER was more favorable when double therapy was studied (see elsewhere in this table)
High systolic BP	Dietary counseling by dietitians to reduce sodium intake (as per current practice in NZ)	NZ$ 36,900 per QALY gained ($NZ 2011) (~ US$ 27,100 in 2018)	The 95%UI for this ICER was reasonably wide at: NZ$ 22,400 to 62,500 [23]
High body-mass index (BMI)	Weight-loss dietary counseling by nurses in primary care	NZ$ 138,000 per QALY gained ($NZ 2011) (~ US$ 101,000 in 2018)	None of the ICERs for particular population groups (e.g., Māori at NZ$ 116,000) were much better [38]. Of note is that Table 2 refers to a program with an individual-level component, i.e., the mass media promotion of smartphone apps for weight loss [39]. See also the comment in Table 2 on physical activity interventions for weight loss
Tobacco use	Exercise counseling to enhance smoking cessation	NZ$ 451,000 per QALY gained ($NZ 2012) (~ US$ 328,000 in 2018)	This ICER was based on the 24-week follow-up data, using a discount rate of 3.5% [37], and is probably the most realistic ICER calculated in this study. As such, this ICER would not be considered cost-effective in the NZ context. Of note are population programs which involve an individual-level component that are estimated to be cost-saving. These are listed in Table 2 and involve smoking cessation counseling (with Quitline promotion) [33] and a program for the promotion of smartphone apps for smoking cessation [34]

*All calculated from a NZ health system perspective, for the lifetime of the studied population, and at a 3% discount rate (unless otherwise stated).

## Discussion

### Main findings and interpretation

This case study analysis showed how CVD risk factor domains could be systematically prioritized using a mix of GBD and local data. It first used GBD data to identify the five major risk factor domains for CVD prevention in NZ. In descending order of importance in terms of health loss, these were: high systolic blood pressure, dietary risk factors, high LDL cholesterol, high BMI and tobacco. But when these risk factor domains were then considered by the size of the health gain and cost-savings from interventions, the top ranking went to the dietary risk factor domain. It had the highest impact on six interventions (the highest one of which was estimated to save 894,000 health-adjusted life years and produce health system cost-savings of US$11 billion). Of note, however, was that the interventions targeting risk factors include non-CVD health gains in the total health gain, emphasizing that CVD prevention programs often extend well beyond CVD per se.

These dietary interventions also produce higher per capita health gain for Māori compared with non-Māori [19], and so could contribute to reducing health inequities. Furthermore, some dietary interventions (i.e., those reducing consumption of ruminant meats and dairy products) could also have the potential co-benefits of reducing greenhouse gas emissions [20] and other harmful impacts of livestock agribusiness (e.g., on erosion and flood risk, and on the quality of recreational and drinking water).

**Fig. 1:**
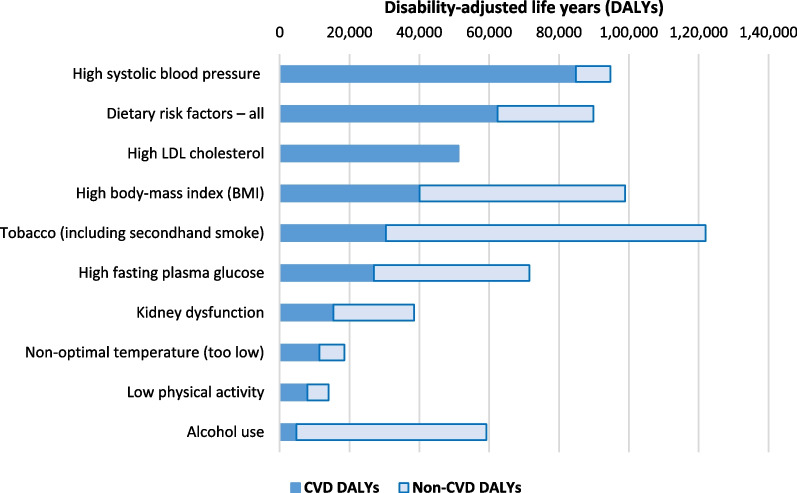
Top 10 risk factors for CVD in NZ ranked by attributable health loss but also showing non-CVD attributable health loss (GBD data for NZ extracted using the GBD Results Tool)

Despite the above, if policy-makers took a broader “total disease” perspective around maximizing the reduction in health loss—then they would potentially prioritize investing in tobacco control above all other risk factors as shown in Fig. 1 (given the additional prevention of other diseases such as cancer and chronic respiratory disease). Such a prominence for tobacco control would coincide with this being a major new area of focus for the NZ Government with recent legislative plans for achieving its Smokefree 2025 Goal [45].

Another consideration for health policy-makers is the strength of evidence for particular interventions—especially with regard to local epidemiology and local health costs. Such evidence for interventions that involve passing new laws (e.g., for enhanced tobacco control or taxing sugar-sweetened beverages) may need particularly high levels of scientific evidence to counter opposition from commercial vested interests.

With all such preventive interventions, a lot will also depend on how they are designed and presented to the public. For example, a tax on sugary drinks has majority public support when it is combined with using the revenue to further subsidize child health in NZ [46]. Nevertheless, some interventions are already likely to be acceptable to a majority of the public, especially if the rationale is well explained. For example, setting maximal sodium levels in products such as bread have been successfully introduced in other high-income countries [47, 48].

### Study strengths and limitations

A strength of this work is that it shows how GBD and local data can be used for prioritization purposes in one high-income country. It also fills a clear gap given that the NZ Government lacks any systematic approach to prioritizing interventions to reduce health loss. Furthermore, this country has relatively high-quality epidemiological and health economic modeling data with BODE^3^ models using consistent approaches. These BODE^3^ model publications have also met the quality inclusion criteria in various systematic reviews (e.g., on sodium [49, 50]; dietary policies [51] and equity [52]). BODE^3^ modeling has also been ranked highest quality out of 25 tobacco control models in a systematic review [53]. But despite these strengths, the following limitations of this study should be noted:

- The GBD Study for risk factor impacts for NZ lack (published) results by ethnicity. Nevertheless, these can be estimated with further epidemiological work if policy-makers requested it, and most of the health economic modeling studies in Table 2 have published results for both Māori and non-Māori (e.g., in a study on prioritizing cancer control interventions [54]).
- The GBD Study might still not be that accurate in some of the risk factor domains. For example, there is still a lot of uncertainty around the precise health harm from air pollution and some recent work produces higher mortality impacts than the GBD Study (e.g., Vohra et al. [55]). Another example is that the strength of evidence for sodium reduction may also have improved since the GBD Study last evaluated it (e.g., from various new studies [56–58]).
- The GBD Study does not include all CVD risk factors. For example, most obvious missing ones include upstream determinants like unemployment [59] and perceived job insecurity [60]. Poverty and socioeconomic inequities may also contribute to CVD in pathways other than the more well-established risk factors considered in Table 1. Various occupational risk factors for CVD are also not included, with these including for NZ: exposures to “dust, smoke or fumes, oils and solvents…” [61].
- The number of health economic studies performed in the different domains in Table 2 may reflect idiosyncratic factors (e.g., research funding and agendas). Nevertheless, many were done by the BODE^3^ Program which purposefully aimed to take a broad approach so as to populate league tables [62], so that policy-makers could be better informed over a wide range of choices.
- Not all the health economic evaluation studies in Table 2 use similar methodologies with this limiting their comparability (this methodological issue for the NZ context is discussed further elsewhere [18]).

### Possible next steps

Given the wealth of methodologically compatible data from the GBD and health economic modeling work for specific countries such as NZ, there is now a need to start operationalizing this information to benefit society by reducing avoidable health loss, reducing health inequities, and making better use of health dollars. For the NZ situation, this may mean that the restructured NZ health system probably needs a specialized unit that focuses on combining epidemiology, health economics and prioritization of health sector interventions. This could be within the proposed Public Health Agency—potentially with the unit also shared with the proposed Māori Health Authority (although the latter could have its own such unit). Alternatively, such a unit could be in a university—with a long-term (e.g., 10-year plus) funding commitment from the central government so that adequate expertise could be established and retained. But failing these developments, it is still possible for officials to use the information in this type of analysis to at least begin incremental moves toward more systematic and rational prioritization that maximizes health gain for the best value for money.

## Data Availability

The dataset supporting the conclusions of this article is included within the article.
